# Irradiated red blood cell transfusion is associated with an increased incidence of deep vein thrombosis in trauma patients: a cohort study

**DOI:** 10.1186/s12871-025-03238-0

**Published:** 2025-08-08

**Authors:** Hua Lu, Li-Fang Wu, Jing-Jing Li, Qi Gao, Gui-Ping Xu

**Affiliations:** 1 Department of Blood Transfusion, the Second Affiliated Hospital of Chongqing Medical University, Chongqing, China; 2 Department of Laboratory Medicine, the Second Affiliated Hospital of Chongqing Medical University, Chongqing, China

**Keywords:** Irradiated red blood cells, Blood transfusion, Deep vein thrombosis, Trauma

## Abstract

**Background:**

Gamma-ray irradiation accelerates the release of red blood cell (RBC)-derived microparticles with procoagulant function, and further promotes the formation of microthrombosis. The use of irradiated RBCs in trauma patients is usually not prohibited. At present, the association that irradiated RBC transfusion and incidence of deep vein thrombosis (DVT) remains unclear.

**Methods:**

This retrospective cohort study included 251 trauma patients between January 2019 and April 2023 at the Second Affiliated Hospital of Chongqing Medical University. The cohort included 147 patients who were only transfused with nonirradiated RBCs (nonirradiation group) and 104 patients who were transfused with irradiated RBCs with or without nonirradiated RBCs (irradiation group). Univariate and multivariate logistic regression analysis was used to analyze the risk factors for DVT.

**Results:**

The incidence of DVT was higher in the irradiation group than in the nonirradiation group (38.5% vs. 22.4%, *p* = 0.006), and irradiated RBC transfusion was associated with an increased incidence of DVT in the univariate analysis (OR 2.16, 95% CI: 1.25–3.77, *p* = 0.006). After adjusting for potential confounders by multivariate analysis, irradiated RBC transfusion appeared to be a risk factor for DVT (OR 2.48, 95% CI: 1.34–4.65, *p* = 0.004). Subgroup analysis revealed that the median storage days of irradiated RBCs and the percentage of irradiated RBCs in total transfused RBCs were positively association with the incidence of DVT (OR 2.95 and 3.19, respectively, both *p* < 0.05).

**Conclusion:**

Irradiated RBC transfusion was associated with an increased incidence of DVT in trauma patients. Irradiated RBC transfusion in trauma patients might need to be reconsidered.

**Supplementary Information:**

The online version contains supplementary material available at 10.1186/s12871-025-03238-0.

## Introduction

Blood transfusion saves thousands of lives every year. However, Blood transfusion has been found to be associated with some adverse outcomes, including multiple organ failure [1, 2], mortality [3, 4], deep vein thrombosis (DVT) [5, 6], and acute lung injury (ALI) [7, 8]. Although the underlying mechanisms of these adverse outcomes are not fully understood, many studies have shown that red blood cell (RBC) storage lesions are an important reason. RBC storage lesions present as lactate accumulation, K^+^ efflux, RBC-derived microparticle (RMP) release, RBC deformation, and so on [9]. As a result, it was found that older RBC transfusion could increase the risk of death [10], multiple organ dysfunction syndrome (MODS) [11], ALI [12], DVT [13], and hospital-acquired infections [14]. However, several large multicenter randomized controlled trials, such as ARIPI [15], ABLE [16], RECESS [17], TRANSFUSE [18], and ABC-PICU [19], compared the use of older RBCs versus fresh RBCs for transfusion and found no significant differences in clinical outcomes among patients. Therefore, the correlation between blood transfusion and adverse outcomes deserves further research.

γ-ray irradiated RBCs are usually used in transplant patients to prevent transfusion-related graft-versus-host disease [20]. However, irradiation can also cause metabolic changes in RBCs, these changes are generally not considered clinically significant, except for patients with renal failure and hyperkalemia [21]. In China, irradiation is mostly carried out by blood centers, and medical institutions usually prestore a certain amount of irradiated RBCs. Because the storage duration of irradiated RBCs is short (14 days) in China, a small amount of irradiated RBCs is also used for nontransplant patients when no transplant patients require them. Furthermore, irradiation-induced storage lesions can also accelerate the release of RMP from RBCs [22, 23]. Studies have shown that RMP can promote the formation of microthrombosis and are closely related to the thrombosis occurring after older RBC transfusion [13, 24]. Therefore; we supposed that irradiated RBC transfusion might increase the risk of DVT.

DVT is more common in patients experiencing trauma, cancer, or major surgery, leading to prolonged patient immobility [25]. At present, a relationship between irradiated RBC transfusion and the incidence of DVT has not been reported. In this investigation, we performed a retrospective cohort study to evaluate the hypothesis that irradiated RBC transfusion is related to the incidence of DVT.

## Materials and methods

### Study design and patient population

This study was approved by the Institutional Review Board of the Second Affiliated Hospital of Chongqing Medical University (SAHCQMU), China (IRB No. 2020178), due to the retrospective nature of the study, informed consent was waived by the Institutional Review Board of the SAHCQMU, and the study complied with the Helsinki Declaration and national regulations (Guideline for the Ethic Review of Biomedical Research Involving Human Subjects). All cases were from the SAHCQMU in China. The institution is a comprehensive tertiary hospital with two branches and does not accept pediatric patients. According to the risk factors of DVT and research objectives [23], those trauma patients were included if they met all of the following criteria: (1) a primary diagnosis of fracture, multiple trauma, fall-related injury, crush injury; (2) mobility limitation necessitating bed rest; (3) hospital stay duration exceeding 3 days. Those trauma patients were excluded if they met one of the following criteria: (1) no RBC transfusion during hospitalization; (2) developed DVT before blood transfusion; (3) pre-hospital bedridden status ≥ 3 days; (4) accompanying malignant tumors; (5) inflammatory bowel disease or systemic lupus erythematosus, which increased the risk of venous thromboembolism.

We collected the information, including patient age, gender, fracture location, type of injury, Glasgow Coma Score (GCS), Injury Severity Score (ISS), blood pressure, laboratory data, proportion of pneumatic compression device, heparin treatment status, procoagulant drugs, hospital days, intensive care unit (ICU) days, in-hospital mortality, RBC storage days, ABO blood group, and total units of transfused RBCs, frozen plasma (FP), cryoprecipitate, and platelets (the analysis was restricted to pre-DVT transfusion exposures). The GCS and ISS were recorded in the medical records by the first contact doctor. We investigated the correlation between blood transfusion and DVT based on blood transfusion recorded between admission and the occurrence of DVT. Two investigators recorded blood product information in the transfusion management system and medical records system; subsequently, both data sets were checked for consistency in the final records to ensure reliability.

### DVT diagnosis

All trauma patients were routinely screened for DVT with ultrasound at admission except unstable critically ill patients, unstable critically ill patients received deferred screening, post-resuscitation evaluation commenced after 24-hour stabilization. Serial D-dimer testing initiated at postoperative day 1 and daily clinical assessments for limb swelling, pain or tenderness on palpation, skin temperature and dark red discoloration, and prominent superficial veins were performed. In postoperative patients with a Caprini score ≥ 5 and persistently elevated D-dimer (3–5 days postoperation), and/or signs of DVT (symptoms such as limb swelling, pain or tenderness, elevated skin temperature and erythema, distension of superficial veins) underwent definitive diagnosis via ultrasonography or contrast venography. DVT was diagnosed by ultrasonography or contrast venography showing solid echoes, filling defects, incomplete closure of the lumen, nonphased blood flow, and significant changes in the diameter of the vascular lumen with the course of the disease.

### RBC storage and utilization

All RBCs were collected by the Chongqing Blood Center, packaged, and stored in a solution containing 3.27 g/L citric acid, 26.3 g/L sodium citrate, 31.9 g/L glucose, 2.22 g/L sodium dihydrogen phosphate, and 0.275 g/L adenine at 2–6 ℃. Irradiated RBCs were prepared by irradiating packed RBCs stored for up to 14 days with 25 Gy γ-rays for 10 min (Biobeam GM8000 irradiator, Eckert & Zieger, Germany). The maximum storage days for nonirradiated RBCs and irradiated RBCs were 35 and 14 days, respectively. Irradiated RBCs were routinely issued to any patient based on stock levels and remaining shelf life. Only transfusion events occurring prior to DVT diagnosis were included in the study.

### Statistical analysis

Quantitative data were presented as the median (interquartile range), while categorical data were presented as the percentage (number). Quantitative variables were first tested for a Gaussian distribution, the data of all variables did not conform to a Gaussian distribution except the hemoglobin data, so an unpaired t test was used to compare hemoglobin values, and the Wilcoxon rank-sum test was used to compare other quantitative variables. The *χ*^2^ test was used to compare categorical data. Variables with *p* < 0.1 in the univariate analysis were subjected to multivariate logistic regression analysis. All analyses were conducted using GraphPad Prism 9 (GraphPad Software, California, USA) and a value of *p* < 0.05 was considered statistically significant.

## Results

### Basic characteristics of case cohort

**Fig. 1:**
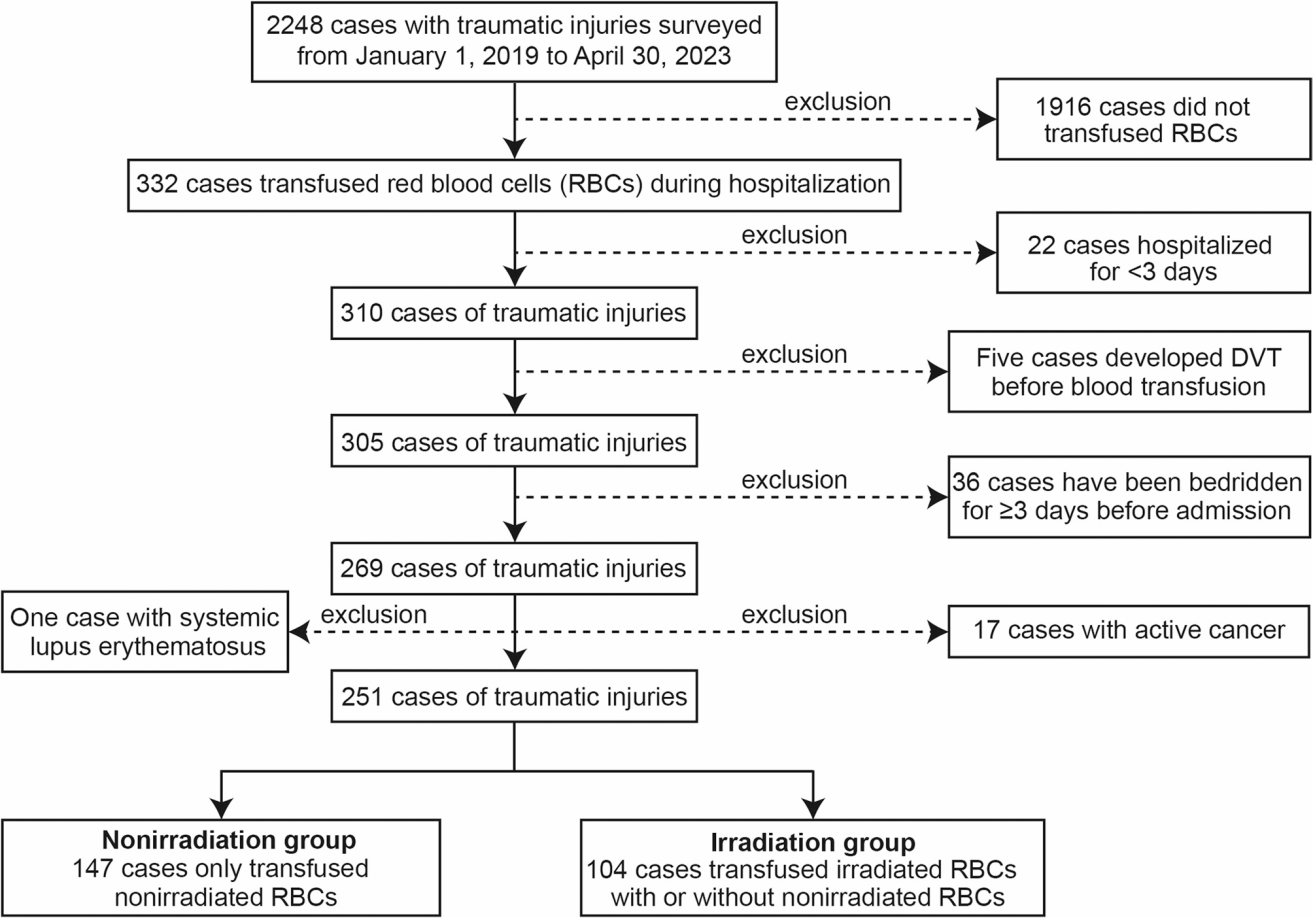
Flow diagram of enrollment process

Overall, this study included 2248 trauma patients between January 1, 2019 and April 30, 2023 at the SAHCQMU in China, of which 1997 patients were gradually excluded according to the exclusive criteria. Finally, 251 trauma patients were included in this study, of which 147 patients received only nonirradiated RBCs (nonirradiation group), while 104 patients received irradiated RBCs with or without nonirradiated RBCs (irradiation group) (Fig. 1). The basic characteristics of 251 patients were summarized in Table 1, there were no statistically significant differences in baseline characteristics, including age, gender, the rate of long bone fracture, the rate of spinal cord injury, the rate of operation, GCS, HCO3^−^ concentration, pH value, activated partial thromboplastin time (APTT), hemoglobin level, the proportion of pneumatic compression device, the proportion of heparin therapy, in-hospital mortality, the maximum storage days of RBCs, the total units of platelet and cryoprecipitate, and the distribution of ABO blood group (Tables 1 and 2). In addition, the incidence of DVT was significantly higher in the irradiation group (40/104, 38.5%) than in the nonirradiation group (33/147, 22.4%) (*p* = 0.006, Table 2). The irradiation group had lower blood pressure, the median storage days of RBCs, and higher ISS, prothrombin time (PT), hospital days, ICU days, the total units of transfused RBCs and FP compared with nonirradiation group (Tables 1 and 2), indicating that patients receiving irradiated RBCs maybe suffer more severe injury, and leading to more severe adverse outcomes. However, there was a low linear correlation between the irradiated RBC transfusion and the indicators of severity of injury, or even other imbalanced factors between study groups (Supplementary Table 1, all,|r| ≤ 0.4, *p* < 0.05). Therefore, the DVT occurrence was at least partially associated with irradiated RBC transfusion.

**Table 1:** Baseline characteristics of patients with trauma

Variables	Nonirradiated (*n* = 147)	Irradiated (*n* = 104)	*p* value
Age	66(49,80)	58(46,71)	0.048
male	54.4%(80)	53.8%(56)	0.928
Long bone fracture	66.7%(98)	65.4%(68)	0.833
Spinal cord injury	8.8%(13)	5.8%(6)	0.364
Surgical operation	93.9%(138)	98.1%(102)	0.109
GCS	15(15,15)	15(14,15)	0.236
ISS	4(3,8)	7(3,22)	0.001
Systolic pressure	126(116,141)	121(105,136.8)	0.016
Diastolic pressure	73(66,80)	68(60.3,78.8)	0.018
HCO3^−^	23.3(20.5,25.3)	22.5(19.9,24.3)	0.126
pH	7.4(7.3,7.4)	7.4(7.3,7.4)	0.202
PT	14.2(13.4,15.7)	14.8(13.8,17.4)	0.004
APTT	37.8(34.2,42.8)	38.3(34.5,42.3)	0.736
Hemoglobin	9.9(8.5,11.3)	10.0(8.1,11.6)	0.617
Pneumatic compression device	89.1%(131)	93.2%(97)	0.261
Heparin therapy	81.6%(120)	87.5%(91)	0.211
Hospital days	20(12,31)	26(16.3,35.8)	0.003
ICU days	0.0(0.0,3.0)	4.0(0.0,8.0)	< 0.001
In-hospital mortality	2.7%(4)	3.8%(4)	0.617

Data presented as median (25th percentile, 75th percentile) or percent (frequency) of patients

*GCS* Glasgow coma score, *ISS* Injury severity score, *PT* Prothrombin time, *APTT* Activated partial thromboplastin time, *ICU* Intensive care unit

**Table 2:** Incidence of DVT and transfusion characteristics in the study cohort

Variables	Nonirradiated (*n* = 147)	Irradiated (*n* = 104)	*p* value
Incidence of deep vein thrombosis	22.4%(33)	38.5%(40)	0.006
Median storage days of RBCs	16.5(12,21)	10.9(6,13.9)	< 0.001
Maximum storage days of RBCs	19(13,24)	18(8,24)	0.097
Total units of RBCs^a^	2.5(2,5)	6(4,10)	< 0.001
Total units of frozen plasma^b^	4(3,6)	9.5(4.3,14.3)	< 0.001
Total units of platelet^c^	1(1,2)	1(1,2)	0.688
Total units of cryoprecipitate	10(10,25)	10(10,10)	0.051
Blood group			
A	32.7%(48)	37.5% (39)	0.427
B	28.6%(42)	24.0% (25)	0.424
O	30.6%(45)	32.7% (34)	0.727
AB	8.2%(12)	5.8% (6)	0.469

Data presented as median (25th percentile, 75th percentile) or percent (frequency) of patients

*RBCs* Red blood cells

^a^RBCs prepared from 200mL whole blood recorded as one unit

^b^100mL plasma recorded as one unit

^c^One therapeutic dose of platelet collected by machine recorded as one unit

### Irradiated RBC transfusion was a risk factor for DVT in trauma patients

**Fig. 2:**
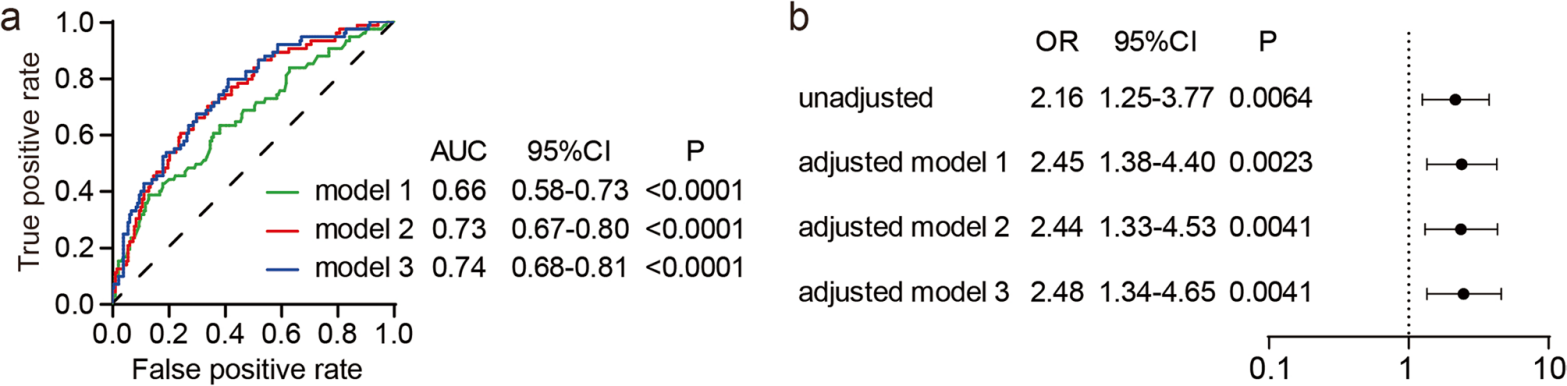
Multivariate logistic regression analysis of the association between irradiated RBC transfusion and the risk of DVT. **a** The ROC curve and AUC were presented in different adjusted models. **b** Irradiation RBC transfusion was associated with the increased risk of DVT in both unadjusted and adjusted models. Model 1: adjusted for age and gender, model 2: adjusted for ISS, diastolic pressure, PT, hip fracture, and tranexamic acid, model 3: adjusted for age, gender, ISS, diastolic pressure, PT, hip fracture, and tranexamic acid. RBC: red blood cell; DVT: deep vein thrombosis; ROC: receiver operating characteristic curve; AUC: area under the ROC curve; OR: odds ratio; CI: confidence interval; ISS: injury severity score; PT: prothrombin time

The variables with a value of *p* ≤ 0.05 in Tables 1 and 2, and those potential confounding factors being associated with DVT occurrence were first analyzed by univariate logistic regression to identify confounders. Results showed that age (OR 1.02, 95% CI: 1.00-1.03, *p* = 0.016), gender (female) (OR 1.80, 95% CI: 1.04–3.14, *p* = 0.036), hip fracture (OR 1.99, 95% CI: 1.07–3.66, *p* = 0.028), PT (OR 0.88, 95% CI: 0.76–0.99, *p* = 0.044), tranexamic acid (OR 2.04, 95% CI: 1.16–3.65, *p* = 0.014) and irradiated RBC transfusion (OR 2.16, 95% CI: 1.25–3.77, *p* = 0.006) were significant association of DVT occurrence, and the ISS and diastolic pressure nearly reached statistical significance (*p* < 0.1, Table 3). Therefore, age, gender, ISS, diastolic pressure, hip fracture, tranexamic acid and PT were included in the subsequent multivariate logistic regression analysis. The results of multivariate logistic regression analysis showed that model 3 (adjusted for age, gender, ISS, diastolic pressure, hip fracture, tranexamic acid and PT) had the highest area under the curve (AUC) (Fig. 2a), and was therefore used as the final model. After adjusting for age, gender, ISS, diastolic pressure, hip fracture, tranexamic acid and PT, irradiated RBC transfusion was still positively associated with the incidence of DVT (OR 2.48, 95% CI: 1.34–4.65, *p* = 0.004) (Fig. 2b). In addition, based on model 3, we further included factors such as ICU days, the total units of transfused RBCs, plasma, and cryoprecipitate, which did not significantly affect the correlation between irradiated RBC transfusion and DVT occurrence (OR 2.55, 95% CI: 1.33–4.91, *p* = 0.005, data not shown).

**Table 3:** Univariate logistic regression for the risk of DVT in trauma patients

Variables	Odds ratio	95% CI	*p* value
Age	1.02	1.00-1.03	0.016
gender, female	1.80	1.04–3.14	0.036
GCS	1.04	0.94–1.17	0.511
ISS	1.02	1.00-1.05	0.070
Brain injury	1.36	0.74–2.46	0.314
Hip fracture	1.99	1.07–3.66	0.028
Systolic pressure	1.01	1.00-1.02	0.257
Diastolic pressure	1.02	1.00-1.04	0.072
PT	0.88	0.76–0.99	0.044
Hospital days	1.00	0.99–1.01	0.734
ICU days	1.00	0.96–1.03	0.915
Prothrombin complex concentrate	0.26	0.01–1.45	0.211
Human lyophilized thrombin powder	1.64	0.21–10.11	0.591
Tranexamic acid	2.04	1.16–3.65	0.014
Median storage days of RBCs	0.98	0.94–1.02	0.349
Maximum storage days of RBCs	1.00	0.97–1.04	0.694
Total units of RBCs	1.01	0.95–1.06	0.788
Total units of FFP	1.00	0.99-1.00	0.638
Total units of cryoprecipitate^a^	0.91	0.76–1.02	0.190
Irradiated RBC transfusion	2.16	1.25–3.77	0.006
Median storage days of nonirradiated RBCs	0.99	0.96–1.03	0.584
Maximum storage days of nonirradiated RBCs	0.99	0.96–1.02	0.549
Total units of nonirradiated RBCs	0.98	0.91–1.05	0.564

*GCS* Glasgow coma score, *ISS* Injury severity score, *PT* Prothrombin time, *ICU* Intensive care unit, *RBC* Red blood cell, *FFP* Fresh frozen plasma, *CI* Confidence interval

^a^Irradiated RBCs prepared from one unit RBCs (approximately 200 mL) recorded as one unit

### The percentage of irradiated RBCs in total transfused RBCs were positively associated with incidence of DVT

We next performed a subgroup analysis in irradiation group. We found that, patients receiving irradiated RBCs with an median storage time of ≥ 6 days had a higher incidence of DVT than patients receiving irradiated RBCs with an median storage time of < 6 days (adjusted OR 2.95, 95% CI: 1.00-8.95, *p* = 0.043, Table 4). And the total units of irradiated RBCs were not significantly associated with DVT occurrence (*p* > 0.05, Table 4). However, the percentage of irradiated RBCs in total transfused RBCs was positively association with the incidence of DVT in both unadjusted and adjusted models (gradually increasing OR, all *p* < 0.05, Table 4).

**Table 4:** Univariate and multivariate logistic regression analysis of the association between the subgroup of irradiated RBCs and the incidence of DVT

		Unadjusted			Adjusted^a^	
Variables	OR	95%CI	P	OR	95%CI	P
Total units of irradiated RBCs						
< 4U	1.00	reference		1.00	reference	
≥ 4U	1.33	0.54–3.08	0.513	1.92	0.70–5.12	0.194
The percentage of irradiated RBCs in total transfused RBCs						
< 20%	1	reference		1	reference	
< 60%	1.99	1.05–3.75	0.034	2.11	1.02–4.39	0.041
≥ 60%	3.05	1.38–6.75	0.006	3.19	1.36–7.53	0.007
Median storage days of irradiated RBCs						
< 6d	1.00	reference		1.00	reference	
≥ 6d	2.43	0.84–7.14	0.099	2.95	1.00-8.95	0.043

*RBCs* Red blood cells, *DVT* Deep vein thrombosis, *OR* Odds ratio, *CI* Confidence interval, *ISS* Injury severity score, *PT* Prothrombin time

^a^Adjusting for age, gender, hip fracture, tranexamic acid, ISS, diastolic pressure, and PT

## Discussion

This article is the first to study the association between irradiated RBC transfusion and the incidence of DVT. A positive correlation was observed between irradiated RBC transfusion and the incidence of DVT, and further subgroup analysis showed that the incidence of DVT increased with the increasing percentage of irradiated RBCs in total transfused RBCs, when the percentage exceeded 20%, the difference was statistically significant. However, transfusion with 100% irradiated RBCs did not appear to further increase the incidence of DVT (40%, 10/25 patients). We did not analyze these data separately because only seven cases were encountered in which the percentage of irradiated RBCs in total transfused RBCs was between > 60% and < 100%, the incidence of DVT was 57.1% (4/7), and this small number of cases might lead to significant statistical bias.

Numerous studies have shown a positive correlation between the volume of RBC transfusion and the risk of venous thrombosis. Some retrospective study reported a dose-dependent association between the volume of RBC transfusion and the risk of vein thrombosis [6, 26]. A prospective study showed a positive correlation between the volume of perioperative RBC transfusion and the incidence of DVT [27]. Our results revealed that the volume of irradiated RBCs or total transfused RBCs was not associated with the incidence of DVT (Tables 3 and 4, both *p* > 0.05). The insignificant effect of the volume of irradiated RBC transfusion on the occurrence of DVT might be due to the dilution effect of nonirradiated RBCs, as the relationship between the volume of nonirradiated RBC transfusion and the occurrence of DVT was not significant (OR 0.98, 95% CI: 0.91–1.05, *p* = 0.564, Table 3). The ≥ 4U subgroup transfused more nonirradiated RBCs than the < 4U subgroup (average 6.06U vs. 4.26U, data not show), and the patients with a low percentage of irradiated RBC transfusion had a lower risk of DVT (Table 4).

The association between older RBC transfusion and the incidence of DVT is still controversial. A retrospective study showed older RBC transfusion had a higher incidence of DVT than young RBC transfusion [13]. However; another prospective study showed no association between RBC storage age and the incidence of DVT [28]. In this cohort, we did not find an association between RBC storage days of total transfused RBCs and the incidence of DVT. Compared with Spinella’s study, our cohort included all transfused patients, whereas Spinella’s cohort included only those patients transfused ≥ 5 RBC units, and the study groups were matched by the amount of RBC transfusion (± 1 unit) [13]. The different results might be attributed to a mixing of RBC storage times or no match in the amount of RBC transfusion. Since adverse effects caused by older RBCs are still in dispute [29], more research is necessary, especially prospective studies. Here, we found that older irradiated RBC transfusion had a higher incidence of DVT than younger irradiated RBC transfusion, which might be due to storage lesions of older irradiated RBCs, leading to the release of more harmful procoagulant substances, such as RMP, but of course, more experiments were needed to confirm this.

Research had shown that the A blood type was an significant risk factor for venous thromboembolism [30], which may be associated with higher plasma VWF levels in individuals with type A blood [31]. While patients with Factor V Leiden with AB blood type had higher risk of venous thromboembolism than those with A, O, and B blood types [32]. However, studies in trauma populations have shown no association between ABO blood type and DVT [13]. Our studies showed that the ABO blood type was not associated with DVT in trauma patients. To date, the association between ABO blood type and DVT remains controversial, and ABO blood type might be associated with DVT in specific disease populations.

Our study was based on a single-center, retrospective case cohort, and there might be some selection biases in the case screening process. Additionally, this sequential analytical approach (univariate followed by multivariate) might overlook certain significant confounding factors. Although surgery might be associated with DVT occurrence, we observed that only 1 out of 73 DVT cases had not undergone surgery (data not shown). When analyzing the association between surgery and the incidence of DVT, the 95% CI was excessively wide (likely due to limited sample size), suggesting imprecise effect estimates. Consequently, Surgery was excluded from the univariate and multivariate logistic regression model, surgery might represent an unadjusted confounder that was not incorporated into the final model. The grouping in Table 4 was designed to ensure similar case numbers across groups to facilitate statistical analysis, this grouping approach involved a degree of subjectivity, and different grouping criteria might lead to slight variations in the results. The maximum limitation of our experiment was that there were fewer patients who received only irradiated RBCs during hospitalization. Setting up a separate group for comparative analysis might result in a large statistical bias. Although the volume and storage time of nonirradiated RBCs showed no correlation with the incidence of DVT, the effects of nonirradiated RBCs in the irradiation group might not be completely excluded by multivariable logistic regression analysis, because as the prolonged storage time, nonirradiated RBCs would also experience storage lesions and RMP release, the effects of storage days and the volume of irradiated RBCs were likely to be masked by nonirradiated RBCs. Another limitation was that some patients were transfused with mixed RBCs that had different storage days; therefore, we could only compare the median storage days, and there might be a large coefficient of variation. A prospective trial that provides patients with RBCs matching the storage age would be better. Furthermore, the irradiated group had greater illness severity (wider ISS range, more transfusions, and longer ICU/hospital stays) than nonirradiated groups, creating potential residual confounding factors beyond statistical adjustment.

## Conclusions

In trauma patients, the incidence of DVT was increased with the transfusion of irradiated RBCs when compared with a group of patients who were transfused nonirradiated RBCs. After adjusting for potential confounding factors there was an independent association between the irradiated RBC transfusion and the incidence of DVT. Subgroup analysis showed that the increased incidence of DVT was associated with the transfusion of irradiated RBCs with prolonged median storage days and high percentage of irradiated RBCs in total transfused RBCs. Our results supported the hypothesis that irradiated RBC transfusion was related to the incidence of DVT. Nevertheless, future prospective randomized controlled trials are necessary.

## Supplementary Information

Supplementary Material 1.

## Data Availability

The raw data analyzed in this study can be obtained upon reasonable request addressed to corresponding author Gui-Ping Xu (guipingxu@cqmu.edu.cn).

## Abbreviations

APTT: Activated partial thromboplastin time
ATP: Adenosine triphosphate
CI: Confidence interval
DVT: Deep vein thrombosis
FP: Frozen plasma
GCS: Glasgow coma score
ICU: Intensive care unit
ISS: Injury severity score
MODS: Multiple organ dysfunction syndrome
MOF: Multiple organ failure
OR: Odds ratio
PT: Prothrombin time
RBC: Red blood cell
RMPs: Red blood cell-derived microparticles
SAHCQMU: The Second Affiliated Hospital of Chongqing Medical University
