# Supplementary material for: Irradiated red blood cell transfusion is associated with an increased incidence of deep vein thrombosis in trauma patients: a cohort study
Source: BMC Anesthesiol. 2025 Aug 8;25:400. doi: 10.1186/s12871-025-03238-0 (PMC12333163; doi:10.1186/s12871-025-03238-0)
Supplement: Supplementary file 1 — Supplementary Material 1. [file 12871_2025_3238_MOESM1_ESM.docx]

**Table S1** Linear correlation analysis between irradiated RBC transfusion and imbalanced factors

| Variables | r | 95% CI | *p* |
| --- | --- | --- | --- |
| Age | -0.12 | -0.25-0.00 | 0.048 |
| ISS | 0.21 | 0.08-0.32 | 0.001 |
| Systolic pressure | -0.15 | -0.27--0.03 | 0.016 |
| Diastolic pressure | -0.15 | -0.27--0.02 | 0.017 |
| PT | 0.18 | 0.06-0.31 | 0.003 |
| Hospital days | 0.19 | 0.06-0.31 | 0.003 |
| ICU days | 0.26 | 0.13-0.37 | <0.001 |
| Total units of RBCs | 0.40 | 0.29-0.50 | <0.001 |
| Total units of frozen plasma | 0.30 | 0.17-0.41 | <0.001 |
| Total units of cryoprecipitate | 0.13 | -0.00-0.25 | 0.046 |

RBCs: red blood cells; CI: confidence interval; ISS: injury severity score; PT: prothrombin time; ICU: intensive care unit. Spearman correlation was used to analyze the linear correlation by Graphpad Prism.
